# Informal caregiving from the perspectives of older people living alone in India

**DOI:** 10.1111/opn.12468

**Published:** 2022-04-24

**Authors:** Lena‐Karin Gustafsson, Ildikó Asztalos Morell, Carl Johansson, Santa De

**Affiliations:** ^1^ School of Health, Care and Social Welfare Mälardalen University Eskilstuna Sweden; ^2^ Department of Urban and Rural Development Swedish University of Agriculture Uppsala Sweden; ^3^ College of Nursing Bharati Vidyapeeth University Pune, Maharashtra India

**Keywords:** community, hermeneutics, informal care, older people, social networking

## Abstract

**Background:**

The cultural and social norms in India stipulate that family and preferably children of the older person, provide the support and care that is needed. In recent years, we have witnessed an overall upsurge in interest in informal care from all countries in the developed world considering their ageing populations. The older people living alone group is, especially interesting in this matter, since it seems to deviate from the expectations of extended family living.

**Objective:**

The aim was to describe older persons' experiences of informal care when living alone in India.

**Methods:**

The study has a hermeneutic design, analysing interviews of older persons living alone in India.

**Results:**

Findings revealed informal care as the thematic patterns: Informal care as a fundamental human responsibility, an obligation and thereby a way to act in ‘common sense’. It was a way of ‘paying‐back’ care that they had received from others in their life history, motivated by governmental care was not presented as an option. Informal care also created safety by the provision of alert and actionable care by loved ones, including spatial safety. Most of the informants experienced themselves as informal caregivers assisting others in need even if they themselves were old and fragile. Providing self care was also seen as a part of informal care conducted by capable and worthy persons. They also pointed out their own obligation to seek informal care and even to listen to the suggestions of younger generations regarding the type and scope of care.

**Conclusions/Implications for practice:**

Informal care in India is not only dependent on having children who ensure that you receive the care you need. Extended family, neighbours and friends feel a basic human obligation to care for the older people in their environment. This responsibility is deeply rooted even within the older people who become fragile in old age.

**What does this research add to existing knowledge in gerontology?**

- Older persons living alone in India view informal care as security, and not in the first instance as the security resources offered by society.
- Informal care is seen here in the Indian context as not only being dependent on having children who ensure that the older persons receive the care they need. Extended family, neighbours and friends seemed to feel a basic human obligation to care for the older people in their environment.

**What are the implications of this new knowledge for the nursing care of older people?**

- The study suggests that the most effective effort that can be made by professional care is to support the already functioning networks of informal care.
- If the care centres have the ambition to reduce loneliness, extra efforts should be made to promote the older persons interaction with non‐professionals.
- In India as well as in Western countries, this is to strengthen older persons existing network that could potentially provide informal care.

**How could the findings be used to influence policy, practice, research or education?**

- The findings can be used by policymakers to increase understanding of the existing needs of older persons in multinational as well as multicultural welfare environments. In the Indian context these findings might be applied on meeting these needs and thereby increase effectiveness of welfare interventions.
- The findings can also be used in educational environments as examples of welfare needs and solutions from a named part of the world. This perspective is becoming increasingly relevant in an increasingly mobile world.
- Meeting these needs could increase effectiveness of welfare interventions in India including gerontological nursing care strategies that serves to support a healthy aging, maximum functioning and good quality of life.

## 1 INTRODUCTION

In this paper, we illuminate a part study from a collaborative research project by researchers from gerontological nursing and social work in India and Sweden. Gerontological nursing aim to, in collaboration with the older adult, their families and rest of the welfare sector support a healthy aging, maximum functioning and good quality of life (CGNA, 2020). In this study, we take our theoretical departure from Eriksson’s (2018) nursing theory and the assumption about informal care or ‘natural care’, as a fundamental human responsibility, experienced as an obligation and thereby a way of acting in ‘common sense’. In Eriksson's theory, she claims that care is based in us within the office of being human and thus natural in all of us, then, the ability develops depending on social and cultural conditions. If we train in care, then, natural care develops into professional care that always contains a core of natural care but more to it. In India, responsibility for care of the older person falls primarily on the family and is performed by informal caregivers that is, unpaid care to someone with whom they have a personal relationship. We refer to this informal care that involves the support of family and friends, as well as the care from the older person him/herself (i.e. self‐care) as natural care. This contrasts to the services of municipal care, privately employed care providers or county council resources, which we call professional care. The viewed responsibility of informal care owes its existence to cultural norms and a lack of formal long‐term care facilities in society (Eliopoulos, 2013). India has the second largest group of older people over 65 years, in the world. The cultural and social norms in India stipulate that the children of the older person, primarily provide the support and care that is needed. In recent years, we have witnessed an overall upsurge in interest in informal care in all countries of the developed world considering their ageing populations and governments’ tight control of welfare expenditure. Therefore, it becomes important to view experiences of a cultural tradition related to care that have a long history for ageing people in India.

### 1.1 Background

WHO (2020) states that between 2015 and 2050, the proportion of the world's population over 60 years will nearly double from 12% to 22%. In India, 7.5% were over 60 years in 2010 (Dey et al., 2012), and in 2020, India is a home to 1.35 billion people where 5.4% are above 65 years (UI: 2020). The growing aging population has increasing social and economic effects, which include higher pension and health care spending, the need for social security reforms, a reduction in the workforce and a shortage of younger people who can support the older persons (Singh et al., 2016). From a health system perspective, this growing number of older people poses complex physical, social and cultural challenges (Abdul Manaf et al., 2016).

A minority (2.4%) of the older people live alone and of those 3.49% are women and 1.42% men (Subhojit et al., 2012). Traditionally, even this segment of older people depends on their children or family for their health and welfare. Social and cultural changes to a more Western lifestyle may challenge this tradition and this kind of support may not be as readily available as it used to (Saxena et al., 2012). Many older persons suffer from multiple morbidities relating to ageing, such as depression, musculoskeletal disorders, hypertension, gastrointestinal problems, diabetes mellitus and neurological problems, that may call for different kinds of care and support (Kamble et al., 2012).

We used Eriksson’s (2002, 2007a, 2018) Theory of Caritative Caring as theoretical foundations of this study. This caring theory differentiates care into natural care and professional care. In this study, we have focused on natural care as the care that is informal and how this was shown among older persons living alone in India. Eriksson (2002, 2018) expresses natural care as something human by nature and is a call to serve in love. The origin of natural care implies cleansing and nourishing, as well as spontaneous and unconditional love. For natural care to work, friendship or human fellowship is required. Eriksson also claims that being willing to serve others is a way of confirming the dignity of oneself. She claims that care is founded in us within the office of being human and thus natural in all of us, then our ability to use that develops depending on social and cultural conditions. Andersson (2013) claims that natural care is part of what makes us human. Rafii et al. (2007) state that natural care occurs spontaneously and without thinking, in contrast to professional care that is reflected on in relation to ethics and evidence.

The view of natural care and the family's responsibility to care for the older people may have cultural differences, depending on traditions, view of gender roles and social affiliation or class. Fink and Lundqvist (2009) argues that welfare interventions in Sweden, Denmark and Great Brittan, have historically been shaped and implemented around the normative idea of the male breadwinner. The ideas of the normative family make for a welfare heritage that to this day influences the gendered inequality in the distribution of welfare services and informal care work in families. That is why we in this study approach family from an empirical perspective, giving the informants space to shape their own definitions of the extension of their families.

A lack of healthcare services prevails in many low and medium‐sized income countries (LMICs) including India. Particularly, older people care supported by the state. This affects the need for informal care, provided by for example, family members of the dependent older person. Relatively little is known about the time that this care demands, its cost and the burden these informal caregivers experience (Brinda et al., 2014). In many Western countries, the role of the informal caregiver is seen as a burden and is described as having well‐known consequences on the informal caregiver's life, physical and mental health, as well as marital and family relationships so called ‘caregiver burden’ (CB; Bevans & Sternberg, 2012). Despite this, Belgian studies show that daughters who are informal caregivers felt it was their duty to take care of their parents. It is seen as a reciprocity principal that the parents looked after them as children; so, parents can expect to be looked after by their children later in life (Lopez Hartmann et al., 2016). The association between perceived social support and subjective caregiver burden in the context of caring for frail older people shows that informal caregivers were dissatisfied with the degree of support and were more likely to experience informal care as a burden (Lopez Hartmann et al., 2019). In Asian countries such as Thailand, informal care by family members is the usual and expected pattern of caregiving in Thai families (Wongsawang et al., 2013). In India, caregiving places great responsibility on family, some may find it to be rewarding while others may feel burdened (Ajay et al., 2017). One Indian study was found that explored informal caregivers’ experiences of the strains of caring for older people and the significant negative correlation with the dependency level of the older people in question. The higher the dependence of the older people, the greater the necessity for evaluation of caregiver strain to ensure the continuum of care for the older people (Jacob et al., 2019). There are more than 5 million dependent older people in India. Old age dependency ratio in India increased to 9.5 (in 2019) per working person from 5.9 (in 1970). There are no government supported care services for the informal care given by family members who are crucial to dependent old parents, and a dearth of research on informal caregiving (Brinda et al., 2014). Informal caregiving demands substantial effort, productive time and the financial resources of caregivers. Caregiver burden worsens the mental health of caregivers. This is particularly marked in women, whose wellbeing has declined over time, and this may in turn lead to poor care for the dependent older parents (Brinda et al., 2014; Gupta et al., 2012). Women, who are usually the primary caregivers, are found to be burdened more due to the ‘expectations’ of caregiving (Srivastava et al., 2016). This is especially the case in rural areas. The time spent on informal caregiving is estimated to be an average of 38.6 h/week (Brinda et al., 2014; Gupta et al., 2012). Despite socioeconomic and sociodemographic transformations, the main support system for the older person continues to be the family (Eliopoulos, 2013). Studies have shown that the most dominant type of living arrangement is multigenerational household, where the older person live up to three generations with spouse, children and grandchildren or with children and grandchildren with a belief that family values are upheld strongly through intergenerational bonds (Samanta, 2014). The rapid increase in the number of older people in India means attention needs to be paid to the factors that contribute to their increased need for health support. Singh et al. (2016), and their factor analytic study, looked at the effects of specific social networks of children, relatives and friends and their effect on health among older adults. Most studies have interviewed or measured relatives’ experiences (Ajay et al., 2017; Bevans & Sternberg, 2012; Brinda et al., 2014; Jacob et al., 2019) or have focussed on employees with informal eldercare responsibilities (Greaves et al., 2015). There is also a lack of studies focusing older persons living alone. We address these limitations and would like to illuminate the older persons’ experiences of informal care living alone in India.

### 1.2 Aim

The aim was to describe older persons’ experiences of informal care when living alone in India.

## 2 METHODS

The method of the study applied a hermeneutic approach inspired by Gadamer’s (1989) philosophy of understanding and interpretation. This approach focuses on the concept's pre‐understanding, pre‐judgement and fusion of horizons that is, during a dialectical movement, a fusion phase when different understandings go together to build new horizons from where we are weaving the world. This emphasises that the informant expressing him/herself and the researcher who tries to understand are connected by a common human consciousness based on the language that makes understanding possible. Hermeneutic text interpretation means that the understanding is always based on pre‐understandings. The professional pre‐understanding in this case is based on the first author's caring science perspective, scientific knowledge, values, prejudices and ethical understanding as well as experience as a gerontological nurse in settings of community care of older persons. The second author's professional pre‐understanding is based on experiences of being a sociologist working in the field of older people living at home in Sweden.

### 2.1 Sample

Participants consisted of seven older persons living alone in India, of which five were women and two men. The older persons were between the ages of 67 and 82. All respondents resided in separate households owned by themselves. Almost all participants were widows/widowers except one who was unmarried. Four respondents were educated to university level among whom one had masters level education and the other three were educated to secondary level (10th level in India). Five respondents were pensioners among whom one had a pension of only Rs.1000/‐ per month and the rest of his expenses were covered by his son, while two of the respondents were non‐pensioners among whom one of the respondents used to manage her finances from the savings of her diseased husband and another participant who used to manage her finances by letting out one of her flats purchased by her diseased husband. After approval from the chief administrative officer in India, the potential interviewees were contacted. All those contacted accepted the invitation.

### 2.2 Data collection

It is important to clarify that participants in this overall research project were a subset of 100 older individuals living at home who were selected for consideration by non‐probability convenient sampling (snowball) technique. Clusters of geographical areas were identified by a principal investigator through a lottery drawn by a neutral individual. About 80 older individuals agreed to participate in the overall project and seven of these fitted the inclusion criteria for this part study aiming to illuminate experiences older persons living alone. The other 73 lived as couples and/or together with their families and were set aside for possible inclusion in other part studies in the overall research project. Three of the interviews were digitally audio taped and four interviews were video recorded. The interviews were conducted by one of the authors who is a native speaking gerontological nursing researcher in India and co‐writer of this paper. Interviews averaged approximately 60–90 min. Data gathered from the interviews were transcribed in the language of communication (Marathi, Hindi and Bengali) and translated into English with forward‐backwords translation by a professional translator together with the research team.

### 2.3 Data analysis

The hermeneutical approach was operationalised in the present study as follows:

*The first step of analysis* was about integrating the text with the readers. To ensure a hermeneutic approach to the text, it was not read or interpreted until the data collection was completed and compiled as a single text. This first step began with an open reading, which means that the researchers asked the text what it had to say about the topic in focus here. In line with Lindwall et al. (2010), the authors’ professional pre‐understanding made the text understandable.

*The second step of analysis* was about fusion of horizons (i.e. what the text says gets in dialogue with the researcher's pre‐understandings). The text was carefully read in relation to the content. The parts of the text that was unfamiliar to the researchers raised new questions. For example, it became obvious that the text talked about how informal care was expected since it creates good karma in those who practice it, which not were expected by the researchers that had a more altruistic view of care.

*The third step of analysis* included putting the new questions, that came up in the second step of analysis, into the text. The following questions were put to the text—What does the text say about the specifics of informal care for older persons in India? What does the text say about the motivation for informal care? There was a movement back and forth in the text to identify quotations that showed the common meaning of the answers.

*The fourth step of analysis* consisted of summarising the understanding of the text in themes that represent the common feature of all experiences. The common feature was formed into four themes with distinctive qualities with two to three subthemes each. Validity of the thematisation was ensured by all the four authors discussing the themes and subthemes together until agreement was reached.

*The fifth and final step of analysis* created a new understanding. Here, the text was read again to reconfirm the thematisation which involved a further abstraction of the themes and an illumination of the new understandings.

### 2.4 Ethical considerations

The study was conducted in accordance with the ethical guidelines of the Declaration of Helsinki (2013), Codex (2017) and was granted ethical approval from the Swedish ethical board on Research, Uppsala, 2019/279. Moreover, in India, ethical approval: BVCON/EC/63/2017. Before the participants gave their consent prior to the interviews, they were given information about the study, their voluntary participation and their right to withdraw from participation without having to give a reason.

## 3 FINDINGS

The findings revealed that the older Indian participants experienced informal care from informal caregivers following the thematic patterns below (Table 1):

**TABLE 1:** Thematisation

Themes	Subthemes
*Informal care as a fundamental human responsibility*	Obligation as a ‘common sense’
	‘Pay‐back’ care
	Governmental care, not an option
*Informal care as creating safety by natural caring relations*	Alert and actionable care providing
	Love as safety
	Spatial safety with help from others
*Identifying themselves as informal caregivers*	To assist others in need even if you yourself are old and fragile
	Providing selfcare
	View of oneself as capable and worthy
*Own obligation to seek the informal care when needed*	To point out when and what help is needed
	Necessary to listen to younger persons’ suggestions

### 3.1 Informal care as a fundamental human responsibility

The specifics of informal care for the older persons interviewed in the study were shown as an obligation to take responsibility for the older person. Daughters‐in‐law, siblings and cousins were obliged to take responsibility, not just the older person's children. Daughters‐in‐law were often expected to stay home and give up their own profession to care for their husband's parents. This was what happened to the informant *F030* when she was young:

He worked in the pharmaceutical company and he said that his mother had made a lot of effort for him from his childhood up to completion of his education. Because his father died when he was small. So, he said that you (the informant) could not go out to work, you should take care of my mother. So, I did the service.

A sense of responsibility towards the dependent older person motivated this type of informal care, as well as an obligation to pay‐back the care the sons or daughters had received as children from their parents. The older persons saw it as natural that authority and control over their situation was in their son's hands, whereas the job of providing care was often assigned to the daughter‐in‐law. Help from society was not mentioned as a possible option by any of the interviewees.

I: Do you have any expectations from society? IP: What expectation should I have? We should carry on as it is. Whom should I expect anything from? I am satisfied with whatever I have. My son takes care of all my needs, I am happy and satisfied with that. I: So families should take care of their senior citizens? IP: Yes, families should look after them or they should make some arrangement for them elsewhere, somewhere not run by the government. F 030

As we can see in the quotation, the older person was open to the possibility of arranging care in other ways if the son was not able to provide the necessary care. However, governmental care was not seen as an option. When other ways of providing care were mentioned, they referred to private non‐professional care, such as a housemaid or similar, outside the sphere of relatives, paid by the son or other relatives.

### 3.2 Informal care as creating safety by natural caring relations

One of the specifics of informal care for the older persons in India was safety. Their wellbeing was secured by the network of people providing them with informal care. The narratives showed that the network provided alert and actionable care leading to an experienced safe situation despite living alone. ‘If there is a serious problem, then I call my younger brother then he immediately comes to help me’. F036. Safety was experienced as being related to their social network including neighbours, siblings and children, and not the security provided by the state or government. Reciprocity of care incorporated even in‐laws, such as daughters/sons‐in‐law. Thus, loving, caring relations gave an experience of security that made help from the state unnecessary: ‘I ‐ Do you have any expectations from society or government? IP‐ All the people love me so there are no expectations. I treat my daughters‐in‐law as my daughters. So, they love me a lot’. F036. The interviews showed that experiencing safety was more about being part of a community than about a single person. Sometimes people who were not related showed care and support in hard times:

So, after the blast incident first I lost my mother and then my daughter too. But my maid used to come and take care with lots of love. Don’t know why she did everything so lovingly. Maybe she felt we had a close bond or maybe her loyalty towards me. F005.

The safety mentioned in the interviews did not always refer to a feeling of being loved and cared for but also of bodily and spatial protection, of avoiding accidents such as malnutrition or bone fractures.

Some days back, when I was not feeling well, one of my brothers fetched meals for me from their house. Now, the current situation is different and has been for the last 2–3 years. From January my health was not good. From that time, I needed more help. I go outside but there must be someone with me. F019

### 3.3 Identifying themselves as informal caregivers

The experience of being more a part of a community than a single person, meant the older people continued to recognise themselves as being helpers even though they were old and fragile. They contributed in diverse ways, depending on their abilities, to assist those in need in their surrounding environment. The older persons provided care to others and in return gained a more meaningful daily life for themselves.

I also listen to other people’s problems. Many people have no one who can listen to their problems. We can’t reduce their problems or share or solve them, but they feel satisfied if at least they talk to someone about it. This is not like counselling. I have not done any course on it. But I listen to them. They also like that I listen to them. People these days don’t have time. People don’t listen. F005

Informal care could be experienced as an important aspect of themselves, established or indicative of who they really were as persons. Informal care was visible in their lives as a never‐ending story from childhood to ageing. The idea of helping others distinguished them as good human beings giving their time to others in need of Informal care:

I will tell you of an incident. When I was travelling by train from our native place to Bombay, there was a pregnant lady sitting beside me. She just told me that she is going to hospital for delivery and there was no one with her to go to the hospital. Then I told her don’t worry about that I will be there with you. So, when she gave birth, I was with her. So, when she delivered, I used to go to give her snacks and meals. Today she keeps in touch and calls me and asks about my wellbeing. F036

Some of the older persons associated informal care as having strong links with their faith and view of life. Informal care was also a way of finding fulfilment and self‐satisfaction. Selfcare also meant they could be a part of their view of themselves as capable and worthy persons.

### 3.4 Their own obligation to seek the informal care when needed

The older persons did not describe natural care as something given to them without them asking for it. They described the importance of asking for help if needed. Others did not have the capacity to guess what was required and could be afraid of going in uninvited and taking over the older person's autonomy. This was also about the surrounding networks feeling secure that the older person really pointed out the help they needed.

I feel that if I call up and say that I am not feeling well, you can come over or bring something for me. They will do it for me. But… You cannot force anybody to help you. How much can a person do for others? Everyone has their own life. (F‐005)

Several of the interviewees explained their urge to stay as independent as possible though all of them knew that they could receive immediate help if they needed it from informal caregivers in their social network.

I don't want to be dependent on anyone, but I take physical help if its needed. My brothers and their children are there for me. They come whenever I need them.! F004.

One of the older persons also described how important it was to listen to younger people's thoughts of how to arrange things. One informant described it as follows: ‘Old people have old thoughts and young people have fresh thoughts. You should listen to young people's ideas of how to handle things’. F010 So, if they needed care, they would also have to adjust to the informal caregivers’ preconditions to deliver care.

## 4 NEW UNDERSTANDINGS AND DISCUSSION

Informal care is a fundamental human responsibility, experienced as an obligation and a way to act in ‘common sense’. Even though the view of informal care and the family's responsibility to care for the older people may have cultural differences, it can be understood as Eriksson (2002) says, as something human by nature and as a call to serve another human being. Considering Eriksson's theory, this is more a universal way of being human than a way of expressing one's culture. Informal care was described in this study, as a way of ‘paying‐back’ care that had been received from others, motivated by the experience that governmental care was not presented as an option. Eriksson (2007a) claims that every human being has a responsibility to himself but also to serve others. We will care because we want to do good but expect no reward for this. In this study, this was seen when the older person did not expect care from a specific person they had cared for earlier, but they expected that others felt the same about serving others. Informal care was also experienced as it created safety as caregivers were alert. Actionable care provided by loved ones included spatial safety. Eriksson (2002) talks about the origin of natural care that implies cleansing and nourishing, as well as spontaneous and unconditional love. However, in one of the quotations in our study, a husband told his wife that she should take care of his mother. One can question if this type of care was a calling of love that Eriksson (2002, 2018) describes, or a calling of duty expected of the woman in her role as a good wife to her husband? Holmgren et al. (2013) claim that one can be squeezed between gendered generational family responsibilities and reversed caring roles caring for older persons. Alluding to gendered generational power structures, the majority of those who care for their older relatives are women, and their involvement is not always a result of their initiative.

The findings here can relate to Gustafsson and Stenberg (2015) in the way that caring became visible in the sense that the caregiver stood up for the frail person's rights. This is what Martinsen et al. (2013) and Eriksson (2007b) describe as ‘areˆte’. Areˆte could be understood by the metaphor of a ‘lioness’, as the caregiver protects the person in their care from not being hurt in any sense. This experienced safety that we see as an expression of informal care could also be understood in the light of the concept ‘advocacy’ (Cole et al., 2014) where the informal caregiver advocates for the older person. Most of the informants experienced themselves as informal caregivers assisting others in need even if they themselves were old and fragile. Providing selfcare was also seen as a practice of natural care viewing themselves as capable and worthy persons. Absolute dignity is, according to Eriksson (2007a), given to every human by creation, and involves the right to be confirmed as unique and worthy. Relative dignity is influenced by society, including human relations. Human dignity also involves the human obligation of serving with love and existing for the sake of others. The older persons also pointed out their own obligation to seek informal care from others, focussing on when it is needed and what is needed. However, the findings show similar descriptions as outlined by Eriksson (2018) that claim people have a responsibility for themselves and their health situation. What Bevans and Sternberg (2012) point out as ‘caregiver burden’ (CB) was not visible in the interviews, neither as a self‐perceived burden, nor as something one was afraid that others would experience in relation to themselves. However, we did not explicitly ask the informal caregivers in this study about CB as Bevans and Sternberg (2012), Wongsawang et al. (2013), Brinda et al., 2014), Lopez Hartmann et al. (2016), Ajay et al. (2017), Jacob et al. (2019) and Lopez Hartmann et al. (2019) did. There was though an illumination of the importance of listening to the younger generation providing care for them.

### 4.1 Discussions of study limitations

One possible limitation of the study could be the fact that it was based on only seven in‐depth interviews, though they were relatively long (up to two hours). Despite this limitation, we suggest that the fact that the informants come from various backgrounds in terms of age, gender and educational and financial resources, means that some sort of saturation of the phenomenon was reached and exceeded in the interviews. The older persons provided us with multiple descriptions of experiences during the interviews. Data could therefore be considered as rich in content and suitable, for what Ricoeur (1976) talked about, the best interpretation of the common meaning of the phenomena in focus. One can also ask if these interpretations can serve as an understanding of informal care by older persons living alone in other kinds of contexts or countries with different cultures, even though qualitative studies according to Polit and Beck (2012) do not have the purpose of generalising their results. This study, however, answers a call for a common need to strengthen the already existing network for older people living alone. The informal care provided includes the members of caregiving networks beyond the primary caregiver and has additional value in the expansive view preferred by participants who witnessed both the delivery and receipt of informal care. It also provided insight into how previous caregiving experiences and interactions influence the older person's safe ageing.

## 5 CONCLUSIONS

This research indicates that informal care in India is not only dependent on having children who ensure that you receive the care you need. Neighbours and friends all seemed to feel a basic human obligation to care for the older people in their environment. This responsibility seemed to be deeply rooted even within the older people themselves, who were happy to help with what they could even if spatial capacity had failed. Informal care is shown here as something experienced as security not in the first place as the security resources offered by society. Informal care does not necessarily rely on the resources of society and is something one has the responsibility to seek as an individual. Perhaps, the most effective effort that Indian society can make is to support already functioning networks of informal care. Supporting social networking centres where the older people can interact with others to strengthen their already existing network is vital and could potentially provide informal care.

This perspective is becoming increasingly relevant in an increasingly mobile world. The findings can be used by policymakers to increase the understanding of the existing needs of older persons in multinational and multicultural welfare environments. Meeting these needs could increase the effectiveness of welfare interventions in India including gerontological nursing care strategies that serves to support a healthy aging, maximum functioning and good quality of life. This can serve as an illumination of the importance to for example, enhance older peoples pre‐existing networks that could potentially provide informal care in India. The findings can also be used in educational environments as examples of welfare needs and solutions from a named part of the world.

## CONFLICT OF INTEREST

No conflict of interest.

## Data Availability

The data that support the findings of this study are available on request from the corresponding author. The data are not publicly available due to privacy or ethical restrictions.
